# Sequence-Based Genotyping of Expressed Swine Leukocyte Antigen Class I Alleles by Next-Generation Sequencing Reveal Novel Swine Leukocyte Antigen Class I Haplotypes and Alleles in Belgian, Danish, and Kenyan Fattening Pigs and Göttingen Minipigs

**DOI:** 10.3389/fimmu.2017.00701

**Published:** 2017-06-16

**Authors:** Maria Rathmann Sørensen, Mette Ilsøe, Mikael Lenz Strube, Richard Bishop, Gitte Erbs, Sofie Bruun Hartmann, Gregers Jungersen

**Affiliations:** ^1^National Veterinary Institute, Technical University of Denmark, Lyngby, Denmark; ^2^International Livestock Research Institute, Nairobi, Kenya

**Keywords:** swine leukocyte antigen, MHC typing, next-generation sequencing, pigs, immunology

## Abstract

The need for typing of the swine leukocyte antigen (SLA) is increasing with the expanded use of pigs as models for human diseases and organ-transplantation experiments, their use in infection studies, and for design of veterinary vaccines. Knowledge of SLA sequences is furthermore a prerequisite for the prediction of epitope binding in pigs. The low number of known SLA class I alleles and the limited knowledge of their prevalence in different pig breeds emphasizes the need for efficient SLA typing methods. This study utilizes an SLA class I-typing method based on next-generation sequencing of barcoded PCR amplicons. The amplicons were generated with universal primers and predicted to resolve 68–88% of all known SLA class I alleles dependent on amplicon size. We analyzed the SLA profiles of 72 pigs from four different pig populations; Göttingen minipigs and Belgian, Kenyan, and Danish fattening pigs. We identified 67 alleles, nine previously described haplotypes and 15 novel haplotypes. The highest variation in SLA class I profiles was observed in the Danish pigs and the lowest among the Göttingen minipig population, which also have the highest percentage of homozygote individuals. Highlighting the fact that there are still numerous unknown SLA class I alleles to be discovered, a total of 12 novel SLA class I alleles were identified. Overall, we present new information about known and novel alleles and haplotypes and their prevalence in the tested pig populations.

## Introduction

The major histocompatibility complex class I molecules (MHC I), encoded by the highly polymorphic MHC class I genes, plays a pivotal role in the cell-mediated immune response against viral infections and cancer, and the knowledge of allele identities is crucial for prediction of vaccine responsiveness and transplantation success. MHC I genes are expressed by most cells and the molecules bind and present antigens of intracellular origin to the circulating MHC I-restricted cytotoxic T lymphocytes (CTL’s) (1, 2). In the entire sequence of the porcine MHC, also referred to as the swine leukocyte antigen (SLA) region, between 7 and 13 classical *SLA I* genes (loci) spanning the centromere of chromosome, 7 have been described (3–5). Of these, only the *SLA-1, SLA-2*, and *SLA-3* genes encode functional proteins with known relevance to CD8 T cell activation. Of the 3 SLA loci, the *SLA-2* allele is transcribed at a higher level than *SLA-1*, and *SLA-3* is the allele with the lowest transcription level (6–8). Although thousands of SLA class I alleles are expected to exist, only 193 classical class I alleles have been identified and officially designated by the International Society for Animal Genetics (ISAG) and the International Union of Immunological Societies (IUIS) Veterinary Immunology Committee (VIC) SLA (ISAG/IUIS-VIC) Nomenclature Committee thus far, including 70 SLA-1, 87 SLA-2, and 36 SLA-3 alleles. All of the SLA alleles and their sequences are published in the SLA section of the immune polymorphism database (IPD)^1^. Despite the importance of SLA class I expression in the induction and maintenance of CTL responses, it is often not comprehensively characterized. This might be explained by the difficulties of analyzing the highly polymorphic SLA class I alleles. Previously, the PCR-SSP typing method using sequence-specific primers (9) was applied in many laboratories. However, the high workload and low specificity, as well as the need for designing novel primers associated with each novel sequence discovered, emphasizes the need for finding new ways to improve SLA class I typing. One very promising method for MHC class I typing is next-generation sequencing (NGS). This technique is already widely used in typing of the human MHC (10–15), and few studies also describe the use of NGS for MHC typing in pigs (7) and primates (16, 17). Different platforms are available and continuously improved, and for this study the Illumina MiSeq platform was chosen due to the low costs and low error rates compared to other NGS platforms (18, 19). Furthermore, NGS of RT-PCR-generated amplicons indicates the level of transcription for each SLA allele, as the normalized number of reads roughly correlate with the relative expression values obtained by quantitative real-time RT-PCR (7).

In this study, we hypothesized that by sequencing barcoded amplicons generated with universal primers, covering as much of the polymorphic SLA class I gene region as possible, we could resolve most SLA class I alleles and detect their transcription levels in a pool of samples, using the Illumina MiSeq system. We examined the SLA class I profile of 72 pigs from four different populations. Overall, 67 different allele sequences were detected, including 12 novel alleles, and 24 haplotypes were observed of which 15 has not been described before. The highest variation in SLA class I profiles was observed in the Danish pig population and the lowest in the Göttingen minipig population. The Göttingen minipig population had the highest level of homozygous animals, which were almost absent in the outbred fattening pigs from Belgium and Denmark. These results provide important information for the use of pigs as large animal models, and the novel allele sequences can be used to expand the neural networks used for prediction of epitope binding to the SLA class I molecules (20, 21).

## Materials and Methods

### Animals

A total of 72 pigs from four different populations were examined for their SLA class I allele diversity and transcription. These included (1) Göttingen minipigs from Ellegaard, Dalmose, Denmark (*N* = 19); (2) Belgian farm pigs (*N* = 30) of the breed Danish Landrace × Pietrain collected from a single production farm by Veterinary and Agrochemical Research Center (CODA-CERVA), Brussels, Belgium; (3) free ranging South West Kenyan pigs (*N* = 10) of unknown breed but probably containing some introgression from an Asian domestication center (22) collected by International Livestock Research Institute (ILRI), Nairobi, Kenya; and (4) Danish pigs (*N* = 13) of the breed Danish Landrace × Yorkshire (LY) with mixed semen from 100% Duroc (D) breeding station boars, leading to 50% LY/50% D (LY/D) crossbreeds collected from a single production farm by National Veterinary Institute (DTU Vet), Frederiksberg, Denmark. This study was carried out after approval from the Danish Animal Experiments Inspectorate under the Ministry of Justice. Kenyan pig sampling procedures were approved by the ILRI animal ethics committee (IACUC), which strictly adheres to guidelines endorsed by UK government legislation.

### Sample Preparation

RNA from the Göttingen minipigs and the Danish pigs was extracted from 2.5 ml whole blood, respectively, and collected into PAXgene blood RNA tubes (PreAnalytiX Cat. no. 762165). RNA from the Belgian pigs was extracted from 1.5 × 10^7^ peripheral blood mononuclear cells (PBMCs). The RNA extraction was done using the PAXgene blood RNA kit (PreAnalytiX, Cat. no. 762174) following the protocol provided by the manufacturer. From the Kenyan pigs, RNA was extracted from PBMC’s kept in RNA later using the high pure RNA isolation kit produced by Roche (Cat#11828665001) according to the manufacturer’s instructions. RNA purity and concentration were measured using a NanoDrop ND-1000 spectrophotometer (Thermo Fisher Scientific). cDNA was synthesized from 0.3 µg/µl purified RNA using oligo(dT) primers and the Quanti-Tect^®^ Reverse Transcription Kit, which also included an initial genomic DNA removal step (Qiagen, Cat. no. 205311). For the Kenyan pigs, the promega reverse transcription system (product # A3500) using oligo(dT) primers was applied, all according to manufacturer’s manual. PCR-SSP was performed as previously described (9) using genomic DNA extracted with the DNeasy^®^ Blood & Tissue Kit (Qiagen, Cat. no. 69504).

### Primer Design and Typing Strategy

#### Primers for Sequencing of Known Sequences

**Figure 1:**
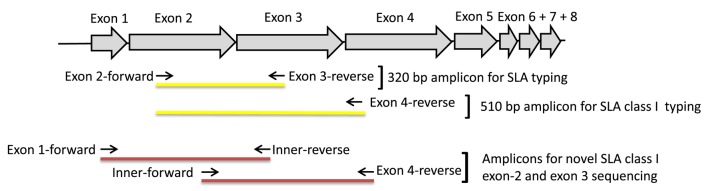
Primer location and amplicon coverage. Schematic view of the SLA class I gene and the location of primers used for generation of amplicons to be sequenced (primer sequences are shown in Table 1).

All known SLA class I coding sequences, kindly provided by Chak-Sum Ho, Gift of Life, Michigan, were aligned, and universal primers were designed in conserved areas of exon 2 and 3 using Primer3,^2^ respectively. These primers amplified 320 nt covering 191 nt of exon 2 (encoding α1) and 129 nt of exon 3 (encoding α2). A 510-nt amplicon covering 191 nt of exon 2 and the complete exon 3 was generated by use of an alternative reverse primer designed in a conserved region of exon 4 (Table 1; Figure 1).

**Table 1:** Primer sequences.

	Forward	Reverse	Product size (bp)
**Primers for SLA class I amplicon generation**			
SLA class I (short)	Exon 2-forward: 5′-CGTGGACGACACGCAGTTC-3′	Exon 3-reverse: 5′-TCCAGYAGCGCAGGTCCTC-3′	320
SLA class I (long)	Exon 2-forward: 5′-CGTGGACGACACGCAGTTC-3′	Exon 4-reverse: 5′-AGGTCAGAGCTGGGGRGG-3′	510

**Primers for sequencing of novel SLA class I sequences (NS)**			
Complete exon 2	Exon 1-forward-SLA-1/3: 5′-GCCCTCTTCCTGCTGCTG-3′	Inner reverse for NS#1–8, 11, 12: 5′-GTAATCGGCGCCGTCGTAGG-3′	420 (*SLA-1 or -3*)
	Exon 1-forward-SLA-2: 5′-AGCCATCCTCATTCTGCTGT-3′	Inner reverse for NS#9: 5′-GTAATCCGCGCCGTCGTAGG-3′	421 (*SLA-2*)
		Inner reverse for NS#10: 5′-GTAATCGGCGCCGTCGTAGC-3′	
Complete exon 3	Inner forward for NS#1, 4, 5, 6, 9, 10: 5′-CGCACAGACTTACCGAGTG-3′	Exon 4-reverse: 5′-AGGTCAGAGCTGGGGRGG-3′	381
	Inner forward for NS#2: 5′-CGCACAGAATTACCGAGTG -3′		
	Inner forward for NS#3,7,8,12: 5′-CGCACAGATTAACCGAGTG-3′		
	Inner forward for NS#11: 5′-CGCAGAGATTAACCGAGTG-3′		

*Primer sequences for generation of the 320 and 510 nt amplicon as well as for sequencing of the complete exon 2 and exon 3 of novel SLA class I sequences are listed. All primers were 5′-barcoded to allow tracing of the fragments to individual pigs. Y = C or T, R = A or G*.

#### Primers for Sequencing of Novel Sequences

To sequence the complete exons 2 and 3 of novel sequences, which is required for the sequences to be approved for the Swine IPD-MHC sequence database, we amplified two regions, one spanning from exon 1 to exon 3 and another spanning from exon 2 to exon 4 for each novel sequence found in the first sequencing run. To amplify the exon 2 spanning amplicon two “outer” forward primers in the conserved areas of exon 1 of *SLA-1* + *3* and *SLA-2*, respectively, were designed and combined with an “inner” reverse primer in the already sequenced area of exon 3 specific for each novel SLA. To amplify the complete exon 3 region an “inner” forward primer specific for each novel sequence was designed in the already sequenced area of exon 2 and combined with an universal “outer” reverse primer in exon 4 (see Figure 1 and Table 1). The two PCR amplicons were sequenced and assembled with the initially found 320 nt sequences for the particular novel SLA sequence discovered, resulting in a 641/642-nt long sequence encoding the complete exon 2 to 3 (Table 1; Figure 1). All primers were tagged with a 5′ barcode of 6 nt and each specific barcode was assigned a specific DNA sample to be able to multiplex all samples for sequencing and subsequently sorting out the sequences for each pig sample. All gene assembly bioinformatics were conducted using the CLC DNA workbench (version 6.0 or 6.5).

### *In Silico* Analysis of Amplicon Sequences

The 320-nt amplicon, covering exon 2 and exon 3 partially, was first analyzed *in silico* to predict the specificity of SLA class I typing based on all known allele sequences, and we found it to resolve 68% of all known alleles specifically, whereas the 510-nt amplicon, covering the complete exon 3 in addition to the 191 nt of exon 2, resolved 88% of all the known alleles completely. The remaining alleles, 32 and 12%, respectively, could be resolved at group-specific allele level. Figure S1 in Supplementary Material shows the coverage of the two amplicons on a molecular 3D structure of a SLA-1 molecule.

### PCR

PCR amplifications were performed using 10 µl 50× diluted cDNA templates in a total of 100 µl containing 1× PCR Gold buffer (Applied Biosystems, Cat. no. N8080246), 1 µM of each primer, 2 mM MgCl_2_, 0.2 mM of each dNTP (Qiagen, Cat. no. 201912), 0.6 µl of AmpliTaq Gold^®^ DNA Polymerase (Applied Biosystems, Cat. no. N8080246), 50 µg/ml Bovine serum albumin, nuclease-free H_2_O, and loading buffer [71 g/ml cresol red (Sigma-Aldrich, Cat. no. 114472-5G), and 15 mg/ml sucrose]. The cycling conditions were 10 min at 94°C, followed by 38 cycles of 20 s at 96°C, 25 s at 62°C, 25 s at 72°C, and then 3 min at 72°C. A fraction of the PCR product was analyzed by gel electrophoresis on E-Gel Agarose Gels, 2% (Invitrogen, Cat no. G6018-02) to ensure correctly sized amplicons. The PCR products were then purified using QIAquick^®^ PCR Purification Kit (Qiagen, Cat. no. 28106), eluted in 80 µl H_2_O, and the DNA concentration and purity was examined using a NanoDrop ND-1000 Spectrophotometer (Thermo Fisher Scientific). The samples were pooled in equimolar concentrations within each sequencing run population group (6 ng/ml for NGS#1 and 10–15 ng/ml for NGS#2).

### Library Preparations

Libraries for NGS#1 were generated based on blunt-end ligation using NEBNext^®^ DNA Library Prep Master Mix Set for 454™ (New England BioLabs, reference nb. E6070). For the NGS#2, the amplicon pool was end-repaired with T4 DNA polymerase, T4 phosphonucleotide kinase, and Klenow fragment enzyme, followed by addition of 3′-end A-overhangs using Klenow 3′–5′exo minus enzyme. Universal Illumina adaptors were ligated on the DNA amplicons using T4 DNA ligase. The ligated PCR amplicon pool was amplified with Herculase II Fusion DNA Polymerase (Stratagene, Agilent Technologies) and primer (PE1 and PE2) with a denaturation time of 2 min at 98°C, followed by 10 cycles of denaturation at 98°C for 30 s, annealing at 65°C for 30 s, and extension at 72°C for 1 min. Final extension was performed at 72°C for 10 min. The spectral profiles corresponding to correct band sizes were verified by visual inspection, and the purity ratio A260/280 of the DNA was measured as between 1.8 and 2. Samples were submitted to NGS facilities where DNA quantity and quality were confirmed on *Qubit*™ fluorometric quantitation (Life technologies) and Agilent 2100 Bioanalyzer using the Bioanalyzer DNA High sensitivity (Agilent Technologies), respectively. The library was denatured with NaOH, diluted with hybridization buffer, and further heat-denatured before running the sequencing. The sequencing was performed with an Illumina MiSeq instrument using paired-end 2 × 300-bp reads and a MiSeq v3 reagent kit for the NGS#2 and the paired-end 2 × 250-bp reads and a MiSeq v2 reagent kit for the NGS#1. The samples were sequenced with addition of 30% PhiX to ensure sequence diversity as described previously (23).

### Sequencing Runs

Two different NGS runs were performed using the Illumina MiSeq platform. The first run, designated NGS#1, was performed at the National High-throughput DNA Sequencing Center at University of Copenhagen on 320 nt amplicons from 29 Belgian pigs, 19 Göttingen minipigs, and 1 Danish pig using the Illumina MiSeq 250 nt paired-end system (250PE). The second run, designated NGS#2, included sequencing of 320 nt amplicons from 10 Kenyan pigs and 12 Danish pigs, and the amplicons for full length assembly of exon 2 and exon 3 from the novel sequences discovered in NGS#1. NGS#2 also included 510 nt amplicons from 20 Belgian pig samples of which 19 were already sequenced using the 320 nt amplicons in NGS#1. The same PBMC RNA extraction from the Belgian pigs was used to generate the two different amplicon sizes. NGS#2 was performed at the DTU Multi-Assay Core, Center for Biological Sequence Analysis, DTU Systems Biology, at the Technical University of Denmark on the Illumina MiSeq with 300 nt paired-end reads (300PE) allowing for longer amplicon sizes.

### Bioinformatics Pipeline

Following sequencing, the reads were processed in a collection of custom Perl scripts. All sequences were first demultiplexed into the individual pig samples according to their barcodes for sequences with both correct barcode and primers. Next, the sequences were cleaned at both ends by removal of bases of a quality less than 98%, which is equivalent to a Phred score of 17. Forward and reverse sequences were then joined, allowing no gaps, a minimum match of 80%, and a minimum overlap length of 35 nt. Each sample was then dereplicated using USEARCH v7.0 (24) into clusters containing 100% identical sequences, and clusters with less than 10 reads were discarded. The consensus sequences of the remaining clusters were now checked for chimeras using UCHIME (25) and the 20 largest non-chimeric clusters were further analyzed using command line BLAST (v2.2.18) with a custom SLA class I library, allowing identities down to 95%, to identify the 100% identical match. Sequences without a 100% identity to any known SLA allele in our own SLA class I library were aligned against GenBank at NCBI using the nucleotide blast task at the NCBI homepage^3^, and if no 100% identical matches were found, the allele sequence was considered as a novel sequence (NS) and further tested for their similarity with all *SLA-1,-2*, or *-3* allele sequences. This was done by constructing a neighbor joining tree from the 605 nt of all novel and known SLA alleles using 1,000 bootstraps replicates. The distances were computed with the Jukes–Cantor method. The analysis was conducted in CLC genomic workbench 6.5.

### Sequencing Outcome

NGS#1: the first sequencing run on the Illumina MiSeq (250PE) generated 29,217,460 reads. Sequences were sorted based on various quality criteria, thereby only maintaining high quality paired-end reads. In the sorting process, 28.44% were removed during demultiplexing, corresponding to the 30% phiX used during library preparations. Additional 4.32% were removed due to barcode errors, which included no room for the barcode, no barcode match, or no barcode. Furthermore, 1.5% was discarded during joining of forward and reverse sequences, and 3.26% were removed during sequence cleaning. Consequently after demultiplexing, mate pair joining, and quality filtering, 8,900,106 paired-end sequences were obtained for further processing. The resulting paired-end sequences were used for clustering using sequence identity of 100%. Most samples were highly dominated by two to six large clusters, which correspond to the expected number of transcribed alleles in each pig sample. The second sequencing run (NGS#2) using the Illumina MiSeq 300PE generated 8,942,478 reads. After demultiplexing, mate pair joining, and quality filtering of the two corresponding result files, a total output read of 490,510 paired-end sequences were obtained for further processing. A large amount of sequences were excluded from NGS#2 due to our stringency in quality control and the higher error rate of the 300PE platform.

The overall quality of both sequencing runs were acceptable, with quality scores ranging from 25 to 40, corresponding to the probability of 1 base call error out of 1,000–10,000 nucleotides. A decrease in sequence quality is observed mainly at the ends of the sequences. The chimera check using UCHIME found chimera sequences in 70 of the 72 pig samples, which were excluded from further analysis. Each sample then included from 3 to 15 clusters, of which 3–6 were typically large and with 100% identity to a known SLA allele and the rest had just above 10 reads and aligned with 99% similarity to one of the already detected sequences, therefore we assume these to be PCR error prone sequences and they were not further analyzed. If one of these typically six or sometimes eight large clusters were not identified as a known allele, they were considered as a novel sequence.

In NGS#1, 66 long alternatively spliced sequences were observed and removed before further analysis. These splice variants were likewise observed by gel electrophoresis as bands or smears showing that it was not a sequencing-generated error. Similar sequences have previously been detected in other studies using NGS-based SLA class I typing (7), and as they contain multiple stop codons in all reading frames, they are considered to be non-functional and were removed before further analysis.

## Results

### SLA Class I Diversity

**Figure 2:**
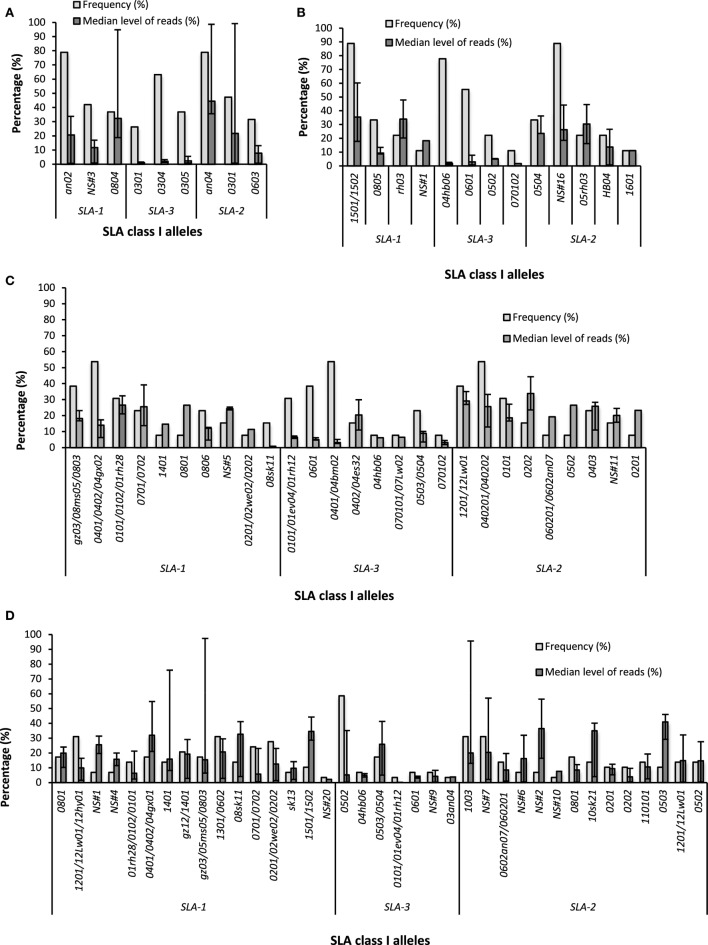
Frequencies and levels of sequencing reads for the SLA class I alleles. The identified SLA class I alleles at each of the three classical SLA class I loci in the four different pig populations are shown; **(A)** Göttingen minipigs (*N* = 19), **(B)** Kenyan pigs (*N* = 9), **(C)** Danish pigs (*N* = 13), and **(D)** Belgian pigs (*N* = 29). For each allele, the frequency of animals having the allele (light gray bars) and level of sequencing reads (dark gray bars) are shown here as median value with error bars indicating range of transcription. The SLA-1*an02 allele could not be verified by the PCR-SSP method.

Among the 72 examined pigs, we identified 50 specific alleles (16 *SLA-1*, 12 *SLA-3*, and 22 *SLA-2*) and 17 group-specific alleles, being alleles that could not be separated from one or two other alleles based on the sequenced part of the allele. Of the latter, 8 *SLA-1*, 6 *SLA-3*, and 3 *SLA-2* were found. Figure 2 shows the frequencies and level of reads for all alleles detected in each pig population. All SLA alleles, sequencing read levels, and haplotype designation for the individual pigs from the four breeds are shown in Tables S1–S4 in Supplementary Material. Haplotypes are defined as sets of *SLA-1, SLA-2*, and *SLA-3* alleles which are observed together in individual homozygous animals or detected together in heterozygous animals of the same breed also expressing another defined haplotype (Tables S1–S4 in Supplementary Material). All detected haplotypes, including their frequencies in the given population, are summarized in Table 2. Seven of the 19 Göttingen minipigs were homozygous; this was only observed for 2/9 Kenyan pigs, and 1/13 Danish pigs, whereas all the Belgian pigs were heterozygous.

**Table 2:** Known and novel haplotypes and their prevalence in the four different pig populations.

Known haplotypes					
**Hp-**	*****SLA-1*****	*****SLA-3*****	*****SLA-2*****	**Found in other breeds, reviewed by Ho et al. (26)**	**Population and prevalence (this study)**

1a.0	*0101*	*0101*	*0101*	Large White	Danish (31%), Belgian (10%, NGS#2 only)
2.0	*0201(a), 0701(b)*	*null*	*0201*	NIH, Sinclair, Hanford	Belgian (17%), Danish (8%)
4b.0	*0401*	*0401*	*040201*	Yucatan	Danish (54%)
6.0	*0805*	*0601*	*0504*	Yucatan, Microminipig	Kenyan (33%)
7.0	*0801*	*070101*	*0502*	Yucatan	Belgian (13.8%), Danish (8%)
17.0	*0804*	*0305*	*0603*	Clawn, Microminipig	Göttingen (37%)
28.0	*0901(a), 1501(b)*	*070102*	*0503*	Landrace	Belgian (10%)
32.0	*0702*	*0402*	*0202*	CMAH/Gal KO	Danish (15%)
62.0	*1401*	*04hb06*	*0602*	Landrace	Belgian (14%), Danish (8%)

**New suggested haplotypes based on this study**					
**Hp-**	*****SLA-1*****	*****SLA-3*****	*****SLA-2*****	**–**	**Population and prevalence (this study)**

A.0	*gz12/1401*	*0502*	*1003*	–	Belgian (28%)
B.0	*gz03/08ms05/0803*	*0601*	*1201/12Lw01*	–	Belgian (14%), Danish (38%)
C.0	*NS#5*	*070102*	*US#11*	–	Danish (15%)
D.0	*0402*	*0503*	*0801*	–	Belgian (17.2%)
E.0	*08sk11*	*0502*	*10sk21*	–	Belgian (10%)
F.0	*1501/1502*	*04hb06*	*NS#16*	–	Kenyan (89%)
G.0	*rh03*	*0601*	*05rh03*	–	Kenyan (22%)
H.0	*HB01*	*0502*	*HB04*	–	Kenyan (22%)
I.0	*0201*	–	*110201*	–	Belgian (7%)
J.0	*NS#20*	*04hb06*	*NS#10*	–	Belgian (7%)
K.0	*0702/0701*	–	*0202*	–	Belgian (7%)
L.0	SLA-1*0806	*0503/0504*	*0403*	–	Danish (23%)
M.0	*1201, 1301*	*0502*	*NS#7*	–	Belgian (28%)
X.0	*an02*	*0304*	*an04*	–	Göttingen (73%)
Z.0	*NS#3*	*0301*	*0301*	–	Göttingen (42%)

*The allele composition of each haplotype is depicted and previously described findings in other breeds included*.

Twelve novel SLA class I alleles were identified as SLA sequences with no matching identity to the (at that time) 155 known SLA class I alleles or sequences in the GenBank at NCBI, and most of these were found in more than one pig. Allele-specific primer sets were designed to obtain full sequences of exon 2 and exon 3 and provided 605/606 nt sequences for 5 of the 10 novel sequences detected in NGS#1, namely, *NS#2, NS#3, NS#7, NS#9*, and *NS#10*. These sequences were then aligned with the corresponding length of all known SLA class I alleles, and phylogenetic analyses were conducted in order to place them at their respective SLA locus. One clustered with the *SLA-1* alleles, three with the *SLA-2* alleles, and one with the *SLA-3* alleles (Figure S2 in Supplementary Material). New IDs were applied to the novel alleles according to their closest relation to the corresponding length of known alleles. All 12 novel SLA class I allele sequences were uploaded to the GenBank at NCBI and assigned an accession number. In addition, five of these allele sequences have been uploaded to the IPD-MHC SLA database and officially designated accordingly (Table 3).

**Table 3:** Novel SLA class I sequences.

Novel sequence #	GenBank accession #	Size (nt)	Exon 2	Exon 3	Closest match	Similarity with closest match (nt)	# and population of pigs expressing the NS	IPD MHC SLA designation
NS#1	KT350995	474	Partial (nt 1–171)	Complete (nt 172–447)	SLA-1*bm04	466/474	3 (1 Kenyan + 2 Belgian)	–
NS#2	KT350996	605	Complete (nt 33–302)	Complete (303–578)	SLA-2*0604	579/605	2 (Belgian)	SLA-2*06:14
NS#3	KT350997	606	Complete (nt 33–302)	Complete (303–578)	SLA-1*sk13	579/606	8 (Göttingen minipigs)	SLA-1*22:01
NS#4	KT350998	473	Partial (nt 1–171)	Complete (nt 172–447)	SLA-1*st11	459/473	2 (Belgian)	–
NS#5	KT350999	282	Partial (nt. 1–171)	Partial (nt 172–282)	SLA-1*0802	279/282	2 (Danish)	–
NS#6	KT351000	426	Complete (nt 33–302)	Partial (nt 303–426)	SLA-2*0901/0902	421/426	2 (Belgian)	–
NS#7	KT351001	605	Complete (nt 33–302)	Complete (nt 303–578)	SLA-2*1001	604/605	9 (Belgian)	SLA-2*10:08
NS#9	KT351002	605	Complete (nt 34–303)	Complete (nt 304–579)	SLA-3*0503	604/605	2 (Belgian)	SLA-3*05:03:03
NS#10	KT351003	605	Complete (nt 33–302)	Complete (nt 303–578)	SLA-2*0601	584/605	2 (Belgian)	SLA-2*06:15
NS#11	KT351004	282	Partial (nt 1–171)	Partial (nt 172–282)	SLA-2*06me01	274/282	2 (Danish)	–
NS#16	KT351008	282	Partial (nt 1–171)	Partial (nt 172–282)	SLA-2*06bm03	273/282	8 (Kenyan)	–
NS#20	KT351012	473	Partial (nt 1–171)	Complete (nt 172–447)	SLA-1*1601	461/473	1 (Belgian)	–

*Table presenting the length of the sequences as well as the part of exon 2 and 3, which were sequenced. Displayed is also the closest matching SLA class I allele and similarity with it. Furthermore, the number of pigs, the population from which each novel sequence was detected along with the ISAG allele designation is listed*.

*–, not designated due to insufficient sequence data*.

In the Göttingen minipigs a total of nine specific SLA class I alleles were identified; three *SLA-1*, three *SLA-3*, and three *SLA-2*. The SLA-1*an02, SLA-3*0304, and the SLA-2*an04 were most prevalent alleles of their respective loci with frequencies of 79, 63, and 79%, respectively. The most abundantly transcribed alleles were the SLA-1*0804 and the SLA-2*an04, whereas the three *SLA-3* alleles had the lowest transcription levels (Figure 2A; Table S1 in Supplementary Material). One novel sequence, *NS#3*, was identified in 8 of the 19 pigs (Table 3). One known haplotype was recognized, Hp-17.0, and two new haplotypes were observed, namely, Hp-X.0 and Hp-Z.0, with Hp-X.0 being the most frequent (Table 2).

In the Kenyan pigs, 13 SLA class I alleles were identified including two novel SLA sequences, *NS#1* and *NS#18*. In addition, two other alleles were not among the 155 alleles in our SLA library; however, using BLAST they were found to be identical with the alleles SLA-1*HB01 (GenBank acc. Nr: AB646190.1) and SLA-2*HB04 (GenBank acc. Nr: AB602434.1) found in Chinese Hebao pigs. The sequencing results of Kenyan pig HB1040 showed one large cluster with 456 nt long sequences. The amplification of a sequence 126 nt longer than the expected size and without any allele variability indicates that the primers do not have an optimal specificity for this pig. Therefore, the following calculations for this population are based on only nine individuals. SLA-1*1501/1502, SLA-3*04hb06, and SLA-2*NS#16 were the overall most prevalent alleles at their respective loci with the frequencies of 89, 78, and 89%, respectively (Figure 2B). These three alleles form a new haplotype called Hp-F.0, which is the most prevalent among the tested pigs from Kenya. The Hp-6.0 haplotype was recognized in three of the nine pigs, and two additional new haplotypes were observed, namely, Hp-G.0 and Hp-H.0, both found in two of the nine pigs (Table 2).

From the Danish fattening pigs, 27 SLA class I alleles were identified, including two novel alleles, and the number of transcribed SLA class I alleles per pig ranged from three to nine with several of the pigs carrying locus duplications. At the *SLA-1* locus, 10 alleles were identified, with SLA-1*0401/0402/04gx02 being the most prominent. Eight *SLA-3* alleles were detected with the most frequent alleles being the SLA-3*0401/04bm02, and nine *SLA-2* alleles were identified of which SLA-2*040201/040202 was observed most frequently (Figure 2C; Table S3 in Supplementary Material). These three alleles are the Hp-4b.0 haplotype, which is found in 54% of the Danish pigs (Table 2). We found three *SLA-1* alleles expressed in four of the pigs, one of them (pig #2007) being a part of the Hp-2.0 haplotype, which is known to have a *SLA-1* locus duplication (SLA-1*0201 and SLA-1*0701). Surprisingly, we also found three *SLA-2* alleles in pig #2041 all transcribed at relatively high levels (11–27% of the SLA class I reads), and pig #2041 and pig #2046 furthermore expressed three *SLA-3* alleles, indicating that *SLA-2*- and *SLA-3* locus duplication may have taken place (Table S3 in Supplementary Material). For pig #2041, with locus duplication at *SLA-1, -2*, and *-3*, the nine transcribed alleles can be divided into two commonly observed haplotypes, namely, Hp-4.0 and Hp-L.0, and haplotype Hp-7.0 only observed in this individual among the Danish pigs (Table 2). Within the Danish fattening pigs, nine known haplotypes were observed; Hp-1a.0, Hp-2.0, Hp-4b.0, Hp-7.0, Hp-32.0, and Hp-62.0, and three new haplotypes were assigned; Hp-B.0, Hp-C.0, and Hp-L.0. Of these, Hp-4b.0 was the most frequent (Table 2).

The allele diversity was also high in the 29 Belgian pigs with 37 alleles identified (16 *SLA-1*, 7 *SLA-3*, and 14 *SLA-2*). Eight novel SLA class I sequences were identified of which one was also found in one Kenyan pig (*NS#1*). The most frequent alleles at their respective loci were SLA-1*1301/0602 and SLA-1*1201/12Lw01/12hy01 (both with a frequency of 31%), SLA-3*0502 (frequency 59%), and SLA-2*1003 and NS#7 (both with a frequency of 31%) (Figure 2D). *SLA-1* gene duplications containing the alleles SLA-1*1201 + SLA-1*1301 or SLA-1*0201 + SLA-1*0701, as previously described for the haplotypes Hp-35.0 and Hp-2.0, respectively, were frequently observed with either of the two duplications observed in 14 of the 29 animals. A few of the animals also expressed more than two *SLA-2* alleles (B4 and F1). For pig B1, multiple alleles were found by NGS#1 which could not be verified by NGS#2 (Table S4 in Supplementary Material). Five known haplotypes were recognized (Hp-1a.0, Hp-2.0, Hp-7.0, Hp-28.0, and Hp-62.0) and eight new were observed (Hp-A.0, Hp-B.0, Hp-D.0, Hp-E.0, Hp-I.0, Hp-J.0, Hp-K.0, and Hp-M.0), with the Hp-A.0 and Hp-M.0 being the most frequent (Table 2). To improve the specificity of the SLA class I typing, we tested the use of a longer amplicon (510 nt) from 20 Belgian pigs of which 19 were repeated from NGS#1. The percentage of the different alleles which could be identified as specific alleles was increased from 65% (24 out of 37) in the samples analyzed using the 320-nt amplicon in NGS#1 to 90% (28 out of 31) in the samples analyzed using the 510-nt amplicon from NGS#2. In spite of the low number of reads per sample in NGS#2, the alleles SLA-2*0101 (expressed in pigs A3, D4, and F5), SLA-3*070102 (expressed in B3, C3, and E4), and SLA-3*070101/07Lw02 (expressed by E3) were detected in this run using primer pair 2, while they were not amplified in NGS#1 using primer pair 1. SLA-3*0101 was detected in NGS#2 in pigs D3, D4, and F5, but only very few reads in pig F2 of NGS#1 (Table S4 in Supplementary Material).

To verify some of the typing results from the NGS-based SLA class I typing, two Göttingen minipigs and two Belgian pigs were SLA class I typed using the PCR-SSP method on genomic DNA. Most alleles were confirmed with both methods except the SLA-1*an02 found in the Göttingen minipig #319871. Furthermore, using the PCR-SSP method, a number of additional PCR products appeared as weak bands when analyzed by gel electrophoresis, which could be an indication of false positives or the presence of silent, non-transcribed alleles (Tables S1 and S4 in Supplementary Material).

## Discussion

Next-generation sequencing of amplicons generated from the transcribed exon 2 and exon 3 of the SLA class I genes is only one of several approaches for MHC class I typing. In pigs, this method has previously provided reliable results for SLA class I typing including transcription levels (7). Besides detection of known alleles, the method also allows for discovery of novel SLA class I sequences, as long as they are similar in the primer binding area of the gene. As many thousand HLA class I alleles have been found in humans, we speculated if it could be possible to find SLA class I sequences not previously described in pigs. A total of 12 novel sequences were detected of which we succeeded to sequence full exon 2 and 3 for five of these. Phylogenetic analysis was conducted with these to determine the closest matching allele(s), indicating what locus the novel sequences occupied. Beyond matching to closest alleles, the phylogenetic tree did not support the locus allocation of all the known SLA alleles. However, when using full length sequences of all known SLA alleles, a correct clustering between *SLA-1, SLA-2*, and *SLA-3* alleles was observed for almost all alleles, although a few alleles still seems to be misplaced (data not shown). This emphasizes the importance of using the complete sequences for phylogenetic analysis. Having the novel alleles sequenced in their full length will confirm their locus of origin and will allow us to explore the possibility of them being *SLA-6* alleles, although they showed no identity with any of these at the NCBI GenBank. The novel sequences were found to differ from already known sequences by only one or a few nucleotides, which could be a product of a PCR-induced error, however, since they were observed in multiple individuals, representing multiple different PCR reactions, we consider this unlikely. An incorrectly sized PCR product was obtained from one of the Kenyan pigs, and consequently no SLA class I sequences were collected from this pig. This might be due to poor primer specificity for this animal, showing that the conserved region, in which the universal primer was designed, is not conserved between all breeds. This may require redesign of these primers in future studies as more SLA class I alleles are discovered.

In this study, we first applied a PCR amplicon of 320 nt and second, to increase the specificity of the typing, a longer amplicon on 510 nt. The large amplicon generally confirmed the results generated with the short amplicon, although, as outlined in Table S4 in Supplementary Material, a few lowly transcribed alleles were detected with NGS#1 and not by NGS#2. Some of these differences can be explained by low sequencing depth of the sequencing run with the long amplicon disabling the detection of some lowly transcribed *SLA-3* alleles, or may be false positives generated through the high number of PCR cycles.

Four of the pigs were typed using the PCR-SSP method to verify typing results from the NGS-based SLA class I typing. Of these, only one allele was found using the NGS but not the PCR-SSP method namely the SLA-1*an02. The reason for this is unclear as this allele was detected by PCR-SSP in the other tested Göttingen minipig proving that the primer pair worked. The PCR-SSP amplification of genomic DNA method furthermore detected some alleles not found using the NGS analysis of transcribed genes, which could be due to absent or very low transcription of, e.g., the SLA-1*08XX found in #319871 but is most likely a product of unspecific PCR-SSP amplification as the PCR product appeared as weak bands when analyzed by gel electrophoresis.

A previous study has showed that the number of reads obtained by pyrosequencing roughly corresponds to the relative quantitative values of the expressed *SLA* genes detected by RT-PCR (7). This is, however, not the only parameter that influences the resulting number of reads as the PCR efficiency may differ between samples. Nevertheless, we found an overall locus-specific transcription pattern of *SLA-2* > *SLA-1*>>*SLA-3* (Tables S1–S4 in Supplementary Material) consistent with the SLA class I transcription levels identified in Clawn and Landrace pedigrees (7) and in the pig aortic endothelial cell line (6) supporting that sequence read levels are an indication of transcription level in most samples.

As SLA class I genes from the three different loci are normally linked, and thereby inherited together, a more simple way to address SLA class I alleles is to assign haplotypes. A total of 24 different haplotypes were observed among the four groups. Only nine of those have previously been assigned to the IPD-MHC database.^4^ The haplotypes Hp-B.0, -1a.0, -2.0, -7.0, and -62.0 were identified in both Danish and Belgian pigs. This is not surprising as they share some genetic background with both populations being 50% Landrace. For the Danish pigs, the haplotype Hp-4b.0 was the most prominent, and this has been described in Yucatan miniature pigs (27). Its low-resolution counterpart Lr-4.0 has previously been identified to be the most prevalent class I haplotype in Danish commercial pigs and in domestic swine across the world (28, 29), which makes this haplotype interesting to consider when designing porcine sub-unit vaccines. The SLA-1*1501/1502 allele found in the Kenyan pigs was previously detected in Landrace (5) and Microminipigs (30), and the SLA-1*HB01 and SLA-2*HB04 alleles have previously been described in Chinese Hebao pigs. This indicates that these pigs originated from European pigs but that genes from Asian pigs have been introduced at some point as suggested by Ramírez et al. (22). In the Göttingen minipigs, two new haplotypes, Hp-X.0 and Hp-Z.0, were observed, including alleles previously described by Ando et al. (31). None of the haplotypes observed in the Göttingen minipigs were found in the other pig populations. This should be considered before using Göttingen minipigs as a model for normal fattening pigs in, e.g., vaccine development studies. Furthermore, the Göttingen minipigs displayed the highest percentage of homozygous individuals. This might be advantageous, e.g., for prediction and verification of CTL responses and uniform responses in immunogenicity studies, but not representative for more outbred pig breeds. Overall, the large variation in haplotypes between different pig populations stresses the importance of SLA class I haplotype considerations when designing vaccines for worldwide use.

*SLA-1* locus duplications have previously been described for the haplotypes Hp-2.0, Hp-28.0, and Hp-35.0 in Sinclair, Hanford, and Landrace pigs (5, 32, 33). These haplotypes, including *SLA-1* duplications, were frequently observed among the Belgian pigs, and furthermore, one of the Kenyan pigs also expressed three *SLA-1* alleles indicating that *SLA-1* duplication had occurred in that haplotype as well. This confirms the prevalence of *SLA-1* locus duplications among different pig breeds. Furthermore, three *SLA-2* and three *SLA-3* alleles were found to be expressed among the Belgian and Danish pig populations, indicating that *SLA-2* and *SLA-3* locus duplication may have taken place. Duplications of the *SLA-2* and *SLA-3* loci have, to our knowledge, not been described earlier, although Tanaka-Matsuda et al. (5) showed 13 and 8 classical SLA class I genes in the Hp-28.0 and Hp-62.0 haplotypes, respectively, showing the large variation in copy numbers between haplotypes. The presence of too many expressed and functional MHC proteins per individual may be an immunological disadvantage due to the process of negative selection during T cell maturation (34). On the other hand, the presence of more than six different MHC class I alleles has been observed among both cattle and sheep (35, 36). This raises the question about how many of these are actually expressed to a biological important level and how well they are capable of binding and presenting antigen to CD8 T cells. For instance, it has recently been indicated that SLA-3 molecules are not as stable binders as SLA-1 and SLA-2 (37) the reason for this is still unknown, but it could suggest that SLA-3 possesses other functions compared to SLA-1 and SLA-2.

Overall, this study takes advantage of NGS to provide new information about SLA class I profiles in four different pig populations and identify novel SLA class I alleles and their inheritance with other alleles, forming previously unknown haplotypes. This information is important whether pigs are used for vaccine development or, as large animal models, for human diseases involving the CD8 T cell-mediated adaptive immune response.

## Ethics Statement

This study was carried out after approval from the Danish Animal Experiments Inspectorate under the Ministry of Justice. Kenyan pig sampling procedures were approved by the ILRI animal ethics committee (IACUC), which strictly adheres to guidelines endorsed by UK government legislation.

## Author Contributions

MRS: study design, experimental setup, sequence analysis, and manuscript preparation. MI: sample preparation and molecular biological work, sequence analysis, and participation in manuscript preparation. MLS: bioinformatic sequence analysis and experimental design. GE and SH: sequence analysis and manuscript preparation. RB: sampling materials from the Kenyan pigs, including RNA extraction and cDNA synthesis. GJ: study design and manuscript preparation.

## Conflict of Interest Statement

The authors declare that the research was conducted in the absence of any commercial or financial relationships that could be construed as a potential conflict of interest.

## Footnotes

^1^http://www.ebi.ac.uk/ipd/mhc/sla/.

^2^http://primer3.ut.ee/.

^3^http://blast.ncbi.nlm.nih.gov/Blast.cgi.

^4^http://www.ebi.ac.uk/ipd/mhc/sla/.
