# Supplementary material for: Sequence-Based Genotyping of Expressed Swine Leukocyte Antigen Class I Alleles by Next-Generation Sequencing Reveal Novel Swine Leukocyte Antigen Class I Haplotypes and Alleles in Belgian, Danish, and Kenyan Fattening Pigs and Göttingen Minipigs
Source: Front Immunol. 2017 Jun 16;8:701. doi: 10.3389/fimmu.2017.00701 (PMC5472656; doi:10.3389/fimmu.2017.00701)
Supplement: Supplementary file 1 [file Table_1.PDF]

Supplementary table 1. SLA class I alleles and haplotypes in Göttingen minipigs (N=19). Number of reads coding for SLA class I molecules are displayed and percentages of these are shown for each allele expressed by the *SLA-1*, *-2*, or *-3* loci as indicated. Novel sequences (NS) were placed in the SLA-1, -2, or -3 columns according to the phylogenetic analysis (supplementary figure 2 and data not shown). Data was obtained in NGS#1. SLA class I typing of pig #319871 and 319938 using the PCR-SSP method is included for comparison. Alleles in brackets appeared as weak bands when analysed by gel electrophoresis.

Animal ID	Reads	SLA-1	%	SLA-3	%	SLA-2	%	Haplotype
319871	141546	SLA-1*an02	31.1	SLA-3*0304	1.5	SLA-2*an04	67.4	Hp-X.0
319871<br>(PCR-SSP)		SLA-1*08XX<br>(SLA-1*11XX)		SLA-3*03XX<br>(SLA-3*0801)		SLA-2*an04<br>(SLA-2*03XX)		?
319938	140603	SLA-1*an02	18.0	SLA-3*0304	3.2	SLA-2*an04	42.7	Hp-X.0
		SLA-1*0804	25.2	SLA-3*0305	2.4	SLA-2*0603	8.4	Hp-17.0
319938<br>(PCR-SSP)		SLA-1*an02/0501<br>SLA-1*08XX<br>(SLA-1*1501)<br>(SLA-1*cs02)<br>(SLA-1*11XX)		SLA-3*03XX<br>(SLA-3*05XX)<br>(SLA-3*0801)<br>(SLA-3*0601)		SLA-2*an04<br>SLA-2*06XX<br>(SLA-2*01XX)<br>(SLA-2*03XX)<br>(SLA-2*w14yn01)<br>(SLA-2*w13sm20)<br>(SLA-2*1601)		(Hp-17.0)<br>?
319751	169997	SLA-1*an02	19.8	ND	-	SLA-2*an04	80.2	Hp-X.0
319774	99368	SLA-1*0804	94.9	SLA-3*0305	4.0	SLA-2*0603	1.1	Hp-17.0
319861	79436	SLA-1*0804	18.7	SLA-3*0305	5.6	SLA-2*0603	13.1	Hp-17.0
		SLA-1*an02	17.4	SLA-3*0304	2.5	SLA-2*an04	42.8	Hp-X.0
319883	126775	SLA-1*an02	20.6	SLA-3*0304	1.7	SLA-2*an04	42.0	Hp-X.0
		NS#3	12.5	SLA-3*0301	1.4	SLA-2*0301	21.7	Hp-Z.0
319907	99752	SLA-1*an02	19.4	SLA-3*0304	1.3	SLA-2*an04	43.7	Hp-X.0
		SLA-1*0804	24.7	SLA-3*0305	1.5	SLA-2*0603	9.3	Hp-17.0
319955	53471	SLA-1*an02	19.9	SLA-3*0304	1.7	SLA-2*an04	44.4	Hp-X.0
		NS#3	11.3	SLA-3*0301	1.1	SLA-2*0301	21.5	Hp-Z.0
320026	165827	SLA-1*an02	31.6	SLA-3*0304	2.5	SLA-2*an04	65.9	Hp-X.0
320067	103547	NS#3	17.0	SLA-3*0301	1.6	SLA-2*0301	22.2	Hp-Z.0
		SLA-1*an02	21.1	SLA-3*0304	2.6	SLA-2*an04	35.5	Hp-X.0
320076	96282	NS#3	13.8	SLA-3*0301	0.2	SLA-2*0301	28.3	Hp-Z.0
		SLA-1* 0804	46.2	SLA-3*0305	4.1	SLA-2*0603	7.4	Hp-17.0
320087	30977	NS#3	1.1	ND	-	SLA-2*0301	98.9	Hp-Z.0

320103	53869	*SLA-1* 0804*	43.0	*SLA-3*0305*	0.8	ND	-	Hp-17.0
		*SLA-1*an02*	2.8	ND	-	*SLA-2*an04*	53.3	Hp-X.0
320145	99665	*SLA-1*an02*	22.6	*SLA-3*0304*	1.3	*SLA-2*an04*	40.7	Hp-X.0
		SLA-1 0804*	32.4	*SLA-3*0305*	2.3	*SLA-2*0603*	0.8	Hp-17.0
320288	106159	*SLA-1*an02*	23.1	*SLA-3*0304*	2.3	*SLA-2*an04*	41.2	Hp-X.0
		NS#3	12.2	*SLA-3*0301*	0.7	*SLA-2*0301*	20.5	Hp-Z.0
320302	140051	*SLA-1*an02*	25.7	*SLA-3*0304*	1.7	*SLA-2*an04*	46.3	Hp-X.0
		NS#3	8.5	ND	-	*SLA-2*0301*	17.9	Hp-Z.0
319818	117347	*SLA-1*an02*	33.8	*SLA-3*0304*	3.0	*SLA-2*an04*	63.2	Hp-X.0
319838	21558	*NS#3*	0.8	ND	-	*SLA-2*0301*	99.2	Hp-Z.0
319981	100831	*SLA-1*an02*	0.6	ND	-	*SLA-2*an04*	98.7	Hp-X.0
		ND	-	ND	-	*SLA-2*0301*	0.7	?
