# Supplementary material for: Sequence-Based Genotyping of Expressed Swine Leukocyte Antigen Class I Alleles by Next-Generation Sequencing Reveal Novel Swine Leukocyte Antigen Class I Haplotypes and Alleles in Belgian, Danish, and Kenyan Fattening Pigs and Göttingen Minipigs
Source: Front Immunol. 2017 Jun 16;8:701. doi: 10.3389/fimmu.2017.00701 (PMC5472656; doi:10.3389/fimmu.2017.00701)
Supplement: Supplementary file 2 [file Table_2.PDF]

Supplementary table 2. SLA class I alleles and haplotypes in Kenyan pigs (N=10). Number of reads coding for SLA class I molecules are displayed and percentages of these are shown for each allele expressed by the *SLA-1*, *-2*, or *-3* loci as indicated. Novel sequences (NS) were placed in the SLA-1, -2, or -3 columns according to phylogenetic analysis (supplementary figure 2 and data not shown). Data was obtained in NGS#2.

**Animal ID**	**Reads**	***SLA-1***	**%**	***SLA-3***	**%**	***SLA-2***	**%**	**Haplotype**
HB 1040	-	ND	-	ND	-	ND	-	-
HB 1052	1981	*SLA-1*0805*	13.4	*SLA-3*0601*	0.7	*SLA-2*0504*	23.6	Hp-6.0
		*SLA-1*1501/ 1502*	40.5	*SLA-3*04hb06*	1.3	*NS#16*	20.5	Hp-F.0
HB 1054	1992	*SLA-1*rh03*	47.8	*SLA-3*0601*	7.7	*SLA-2*05rh03*	44.5	Hp-G.0
HB 1055	2902	*SLA-1*1501/ 1502*	38.7	*SLA-3*04hb06*	2.5	*NS#16*	44.1	Hp-F.0
		*SLA-1*HB01*	23.0	*SLA-3*0502*	4.9	*SLA-2*HB04*	0.7	Hp-H.0
HB 1056	2672	*SLA-1*1501/ 1502*	60.2	*SLA-3*04hb06*	1.3	*NS#16*	25.7	Hp-F.0
		ND	-	*SLA-3*070102*	1.7	*SLA-2*1601*	11.0	?
HB 1058	950	*SLA-1*1501/ 1502*	32.2	*SLA-3*04hb06*	1.9	*NS#16*	26.8	Hp-F.0
		*SLA-1*rh03*	20.2	*SLA-3*0601*	2.6	*SLA-2*05rh03*	16.2	Hp-G.0
HB 1059	1818	*SLA-1*0805*	9.3	ND	-	*SLA-2*0504*	36.2	Hp-6.0
		*SLA-1*1501/ 1502*	17.7	ND	-	*NS#16*	18.5	Hp-F.0
		NS#1	18.3					
HB 1066	521	*SLA-1*0805*	8.6	*SLA-3*0601*	3.3	*SLA-2*0504*	23.6	Hp-6.0
		*SLA-1*1501/ 1502*	25.5	*SLA-3*04hb06*	2.3	*NS#16*	36.7	Hp-F.0
HB 1069	3208	*SLA-1*1501/ 1502*	58.1	*SLA-3*04hb06*	1.0	*NS#16*	40.9	Hp-F.0
HB 1074	3063	*SLA-1*1501/ 1502*	32.2	*SLA-3*04hb06*	2.0	*NS#16*	23.2	Hp-F.0
		*SLA-1*HB01*	10.8	*SLA-3*0502*	5.3	*SLA-2*HB04*	26.6	Hp-H.0
