# Supplementary material for: Sequence-Based Genotyping of Expressed Swine Leukocyte Antigen Class I Alleles by Next-Generation Sequencing Reveal Novel Swine Leukocyte Antigen Class I Haplotypes and Alleles in Belgian, Danish, and Kenyan Fattening Pigs and Göttingen Minipigs
Source: Front Immunol. 2017 Jun 16;8:701. doi: 10.3389/fimmu.2017.00701 (PMC5472656; doi:10.3389/fimmu.2017.00701)
Supplement: Supplementary file 3 [file Table_3.PDF]

Supplementary table 3. SLA class I alleles and haplotypes in Danish pigs (N=13). Number of reads coding for SLA class I molecules are displayed and percentages of these are shown for each allele expressed by the *SLA-1*, *-2*, or *-3* loci as indicated. Novel sequences (NS) were placed in the SLA-1, -2, or -3 columns according to the phylogenetic analysis (supplementary figure 2 and data not shown). Data was obtained in NGS#2.

Animal ID	Reads	SLA-1	%	SLA-3	%	SLA-2	%	Haplotype
2035	1650	SLA-1*0101/0102/01rh28	27.5	SLA-3*0101/01ev04/ 01rh12	5.7	SLA-2*0101	16.7	Hp-1a.0
		SLA-1*0401/0402/04gx02	13.7	SLA-3*0401/04bm02	3.2	SLA-2*040201/040202	33.2	Hp-4b.0
2043	9956	SLA-1*0401/0402/04gx02	15.0	SLA-3*0401/04bm02	3.8	SLA-2*040201/040202	24.8	Hp-4b.0
		SLA-1*gz03/08ms05/0803	23.1	SLA-3*0601	6.1	SLA-2*1201/12Lw01	27.2	Hp-B.0
2042	4905	SLA-1*0701/0702	25.6	SLA-3*0402/04es32	10.9	SLA-2*0202	23.5	Hp-32.0
		SLA-1*1401	14.7	SLA-3*04hb06	6.2	SLA-2*060201/ 0602an07	19.2	Hp-62.0
2038	8919	SLA-1*0701/0702	39.2	SLA-3*0402/04es32	29.9	SLA-2*0202	44.3	Hp-32.0
2045	5480	SLA-1*0101/0102/01rh28	21.0	SLA-3*0101/01ev04/ 01rh12	7.2	SLA-2*0101	27.1	Hp-1a.0
		SLA-1*0401/0402/04gx02	14.0	SLA-3*0401/04bm02	5.1	SLA-2*040201/040202	25.6	Hp-4b.0
2041	6946	SLA-1*0401/0402/04gx02	6.2	SLA-3*0401/04bm02	2.5	SLA-2*040201/040202	12.8	Hp-4b.0
		SLA-1*0801	26.5	SLA-3*070101/ 07Lw02	6.4	SLA-2*0502	26.5	Hp-7.0
		SLA-1*0806	4.6	SLA-3*0503/0504	3.5	SLA-2*0403	10.9	Hp-L.0
2033	3869	SLA-1*0806	12.7	SLA-3*0503/0504	10.1	SLA-2*0403	28.3	Hp-L.0
		SLA-1*gz03/08ms05/0803	16.6	SLA-3*0601	4.7	SLA-2*1201/12Lw01	26.9	Hp-B.0
		SLA-1*08sk11	0.8					
2107	5373	SLA-1*0806	12.1	SLA-3*0503/0504	8.9	SLA-2*0403	25.9	Hp-L.0
		SLA-1*gz03/08ms05/0803	18.1	SLA-3*0601	5.0	SLA-2*1201/12Lw01	29.2	Hp-B.0
		SLA-1*08sk11	0.9					
2046	6124	SLA-1*0101/0102/01rh28	32.4	SLA-3*0101/01ev04/ 01rh12	6.7	SLA-2*0101	16.6	Hp-1a.0
		SLA-1*0401/0402/04gx02	17.3	SLA-3*0401/04bm02	3.2	SLA-2*040201/040202	23.6	Hp-4b.0
				SLA-3*04an07	0.2			
2034	1179	SLA-1*0101/0102/01rh28	25.7	SLA-3*0101/01ev04/ 01rh12	6.3	SLA-2*0101	20.5	Hp-1a.0
		SLA-1*0401/0402/04gx02	15.0	SLA-3*0401/04bm02	5.0	SLA-2*040201/040202	27.5	Hp-4b.0
2040	9416	NS#5	23.5	SLA-3*070102	4.4	NS#11	15.8	Hp-C.0
		SLA-1*gz03/08ms05/0803	18.2	SLA-3*0601	4.7	SLA-2*1201/12Lw01	33.4	Hp-B.0
2037	3890	SLA-1*0401/0402/04gx02	11.1	SLA-3*0401/04bm02	2.6	SLA-2*040201/040202	26.3	Hp-4b.0
		SLA-1*gz03/08ms05/0803	19.8	SLA-3*0601	5.2	SLA-2*1201/12Lw01	35.1	Hp-B.0
2007	78477	NS#5	25.3	ND	-	NS#11	24.5	Hp-C.0
		SLA-1*0201/02we02/0202<br>SLA-1*0701/0702	11.3<br>13.7	SLA-3*070102	1.9	SLA-2*0201	23.3	Hp-2.0
