# Supplementary material for: Sequence-Based Genotyping of Expressed Swine Leukocyte Antigen Class I Alleles by Next-Generation Sequencing Reveal Novel Swine Leukocyte Antigen Class I Haplotypes and Alleles in Belgian, Danish, and Kenyan Fattening Pigs and Göttingen Minipigs
Source: Front Immunol. 2017 Jun 16;8:701. doi: 10.3389/fimmu.2017.00701 (PMC5472656; doi:10.3389/fimmu.2017.00701)
Supplement: Supplementary file 4 [file Table_4.PDF]

Supplementary table 4. SLA class I alleles Belgian pigs (N=30). Number of reads coding for SLA class I molecules are displayed and percentages of these are shown for each allele expressed by the *SLA-1*, *-2*, or *-3* loci as indicated. Novel sequences (NS) were placed in the SLA-1, -2, or -3 columns according to the phylogenetic analysis (Supplementary figure 2 and data not shown). SLA class I haplotypes are displayed in the right column. SLA class I typing of pig A1 and A3 using the PCR-SSP method is included for comparison. Pigs typed using both the long and the short amplicon are marked with *. In bold are the alleles detected with the long amplicon but not the short amplicon. Alleles detected with the short amplicon (NGS#1) but not found with the long amplicon (NGS#2) are marked with #.

Animal ID	Reads	SLA-1	%	SLA-3	%	SLA-2	%	Haplotype
A1*	58557	SLA-1*0801	10.1			**SLA-2*0502**	-	Hp-7.0
		SLA-1*1201/12Lw01/<br>12hy01<br>SLA-1*1301/0602	5.7<br>27.1	**SLA-3*0502**		NS#7	57.0	Hp-M.0
A1<br>PCR-<br>SSP		SLA-1*08XX<br>SLA-1*12XX<br>SLA-1*13XX/06XX		SLA-3*05XX		SLA-2*05XX		?
A2	55468	NS#1	31,4	NS#9	0.3	NS#2	56.4	?
		NS#4	11,4	ND	-	NS#6	0,4	?
A3*	71660	SLA-1*01rh28/ 0102/0101	9,6	ND	-	**SLA-2*0101**	-	Hp-1a.0
		SLA-1*0401/0402/04gx01	48,7	SLA-3*0503/0504	33.2	SLA-2*0801	8.5	Hp-D.0
A3<br>PCR-<br>SSP		SLA-1*01XX<br>SLA-1*04XX<br>SLA-1*1501		SLA-3*05XX<br>SLA-3*01XX		SLA-2*01XX<br>SLA-2*w08XX		?
A4*		SLA-1*0401/0402/04gx01	32	SLA-3*0503/0504	25,9	SLA-2*0801	11.7	Hp-D.0
	111077	SLA-1*1401	9.7	#SLA-3*04hb06	6,1	SLA-2*0602an07/<br>060201	14.4	Hp-62.0
A5*		SLA-1*0201/0202/02we02<br>SLA-1*0701/0702	10.3<br>23.1	ND	-	SLA-2*0201	9.4	Hp-2.0
	51272	SLA-1*gz12/1401	2.8	SLA-3*0502	35,2	SLA-2*1003	19.1	Hp-A.0
B1*	36989	SLA-1*0201/0202/02we02<br>SLA-1*0701/0702	11,9<br>11,2			SLA-2*0201	12,4	Hp-2.0
		SLA-1*gz12/1401	13,8	#SLA-3*0502	7.0	SLA-2*1003	12,9	Hp-A.0
		#SLA-1*gz03/05ms05/0803	6,4			#NS#7	2.1	
		#SLA-1*1301/0602	6,7			#SLA-2*10sk21	4.0	

		#SLA-1*1201/12Lw01/<br>12hy01	1.6			#SLA-2*110101	5.9	
		#SLA-1*08sk11	4.1					
B2*	128335	SLA-1*gz12/1401	29.0	#SLA-3*0502	5,4	SLA-2*1003	14,6	Hp-A.0
		SLA-1*gz03/05ms05/0803	14.4	SLA-3*0601	4.3	SLA-2*1201/<br>12Lw01	32.2	Hp-B.0
B3*	92218	SLA-1*0401/0402/04gx01	21,0	SLA-3*0503/0504	5,8	SLA-2*0801	9.5	Hp-D.0
		SLA-1*1501/1502	34.5	**SLA-3*070102**	-	SLA-2*0503	29.2	Hp-28.0
B4	128229	SLA-1*08sk11	37,4	SLA-3*0502	18.8	SLA-2*10sk21	34.3	Hp-E.0
		SLA-1*1401	8,1			SLA-2*0602an07/<br>060201	1	Hp-62.0
						SLA-2*0202	0.4	?
B5*	62007	#SLA-1*0801	24.1	ND	-	SLA-2*0502	27.6	Hp-7.0
		SLA-1*1201/12Lw01/12hy01<br>SLA-1*1301/0602	9,9<br>17.4	#SLA-3*0502	5,1	NS#7	15,8	Hp-M.0
C1*	91956	SLA-1*0801	18.1	**SLA-3*070101<br>/07Lw02**	-	SLA-2*0502	25,4	Hp-7.0
		SLA-1*1201/12Lw01/12hy01<br>SLA-1*1301/ 0602	9,8<br>20,7	SLA-3*0502	5,6	NS#7	20,3	Hp-M.0
C2	87433	SLA-1*gz03/ 05ms05/0803	97.4	ND	-	SLA-2*1201/<br>12Lw01	0.3	Hp-B.0
		SLA-1*01rh28/ 0102/0101	2.3	ND	-	ND	-	?
C3*	137656	SLA-1*0401/ 0402 /04gx01	25	SLA-3*0503/0504	5,1	SLA-2*0801	0,3	Hp-D.0
		#SLA-1*1501/1502	28,7	**SLA-3*070102**	-	SLA-2*0503	41.0	Hp-28.0
C4*	144305	SLA-1*1401	22.0	SLA-3*04hb06	3.7	SLA-2*0602an07<br>/060201	19.6	Hp-62.0
		SLA-1*gz12/1401	24,6	SLA-3*0502	12,6	SLA-2*1003	17.5	Hp-A.0
C5*	112004	SLA-1*0201/02we02/0202<br>SLA-1*0701/0702	14.1<br>1.7	ND	-	**SLA-2*0201**		Hp-2.0
		**SLA-1*gz12/1401**	-	SLA-3*0502	0.8	SLA-2*1003	83.4	Hp-A.0
D1	67057	SLA-1*08sk11	28.0	SLA-3*0502	4.6	SLA-2*10sk21	35.7	Hp-E.0
		SLA-1*gz03/05ms05/0803	31.4	ND	-	SLA-2*1201/<br>12Lw01	0,3	Hp-B.0

D2	107353	*SLA-1*0201/02we02/0202*<br>*SLA-1*0701/0702*	23,1<br>14,3	ND	-	*SLA-2*0201*	5,2	Hp-2.0
		*SLA-1*gz12/1401*	4,6	*SLA-3*0502*	7,5	*SLA-2*1003*	45,2	Hp-A.0
D3*	120281	*SLA-1*0201/02we02/0202*	13,1	***SLA-3*0101***	-	*SLA-2*110101*	19.3	Hp-I.0
		*SLA-1*1201/12Lw01/*<br>*12hy01*<br>*SLA-1*1301/0602*<br>*#SLA-1*sk13*<br>*#SLA-1*wy06*	13,5<br>22.4<br>0.1<br>0.1	*SLA-3*0502*	2,4	*#NS#7*	28,9	Hp-M.0
D4*	71314	*SLA-1*01rh28/0102/0101*	21,3	***SLA-3*0101***	-	***SLA-2*0101***	-	Hp-1a.0
		*SLA-1*1401*	76,0			*SLA-2*0602an07*<br>*/060201*	2.7	Hp-62.0
D5	83036	*SLA-1*0201/02we02/0202*<br>*SLA-1*0701/0702*	3,9<br>0.5			ND	-	Hp-2.0
		ND	-			*SLA-2*1003*	95.6	?
E1*	96025	*SLA-1*0801*	19,9	ND	-	*SLA-2*0502*	0.5	Hp-7.0
		*SLA-1*1201/12Lw01/12hy01*<br>*SLA-1*1301/0602*	8,6<br>29,5	SLA-3*0502	0,3	*NS#7*	41.3	Hp-M.0
E2	72757	*NS#1*	20.0	*NS#9*	8.2	*NS#6*	32,0	?
		NS#4	19.6	*SLA-3*03an04*	3.8	*NS#2*	16,4	?
E3*	62543	*SLA-1*0801*	20.5	***SLA-3*070101***<br>***/07Lw02***	-	*SLA-2*0502*	4,0	Hp-7.0
		*SLA-1*1201/12Lw01/12hy01*<br>*SLA-1*1301/0602*	16,4<br>22,3	SLA-3*0502	2.4	*NS#7*	34,4	Hp-M.0
E4*	95291	*SLA-1*1501/1502*	44.3	***SLA-3*070102***	-	*SLA-2*0503*	46,1	Hp-28.0
		NS#20	2.1	***SLA-3*04hb06***	-	*NS#10*	7.5	Hp-J.0
E5	100875	ND	-	*SLA-3*0502*	2.3	*SLA-2*1003*	82,3	Hp-A.0
		*SLA-1*0702/0701*	5.7	ND	-	*SLA-2*0202*	9,6	Hp-K.0
F1	134799	*SLA-1*08sk11*	41,2	ND	-	*SLA-2*10sk21*	40.2	Hp-E.0
		*SLA-1*1201/12Lw01/12hy01*<br>*SLA-1*1301/0602*	1,7<br>2.8	*SLA-3*0502*	1.1	*NS#7*	3.3	Hp-M.0
		*SLA-1*0702/0701*	2.1	ND	-	*SLA-2*0202*	3.9	Hp-K.0
		*SLA-1*0201/02we02/0202*	1,4	ND	-	*SLA-2*110101*	2.4	Hp-I.0
F2*	53880	*SLA-1*0201/02we02/0202*	14.5	ND	-	*SLA-2*110101*	15.1	Hp-I.0

		SLA-1*1201/12Lw01/12hy01<br>SLA-1*1301/0602	12.8<br>13.2	#SLA-3*0502	5.4	NS#7	19.7	Hp-M.0
		#SLA-1*sk13	19.2	#SLA-3*0101/ 01ev04/01rh12	0.1	**SLA-2*0202/02we02**	-	?
F3	80700	SLA-1*gz12/1401	28.3	SLA-3*0502	4.0	SLA-2*1003	20.0	Hp-A.0
		SLA-1*gz03/05ms05/0803	15.4	SLA-3*0601	3.0	SLA-2*1201/ 12Lw01	29.3	Hp-B.0
F5*	103587	SLA-1*0401/0402/04gx01	54.7	SLA-3*0503/0504	41.3	SLA-2*0801	1.0	Hp-D.0
		SLA-1*0101/0102/01rh28	3.0	**SLA-3*0101**	-	**SLA-2*0101**	-	Hp-1a.0
