# Supplementary material for: Sequence-Based Genotyping of Expressed Swine Leukocyte Antigen Class I Alleles by Next-Generation Sequencing Reveal Novel Swine Leukocyte Antigen Class I Haplotypes and Alleles in Belgian, Danish, and Kenyan Fattening Pigs and Göttingen Minipigs
Source: Front Immunol. 2017 Jun 16;8:701. doi: 10.3389/fimmu.2017.00701 (PMC5472656; doi:10.3389/fimmu.2017.00701)
Supplement: Supplementary file 5 [file Image_1.PDF]

320 bp amplicon coverage   510 bp amplicon coverage

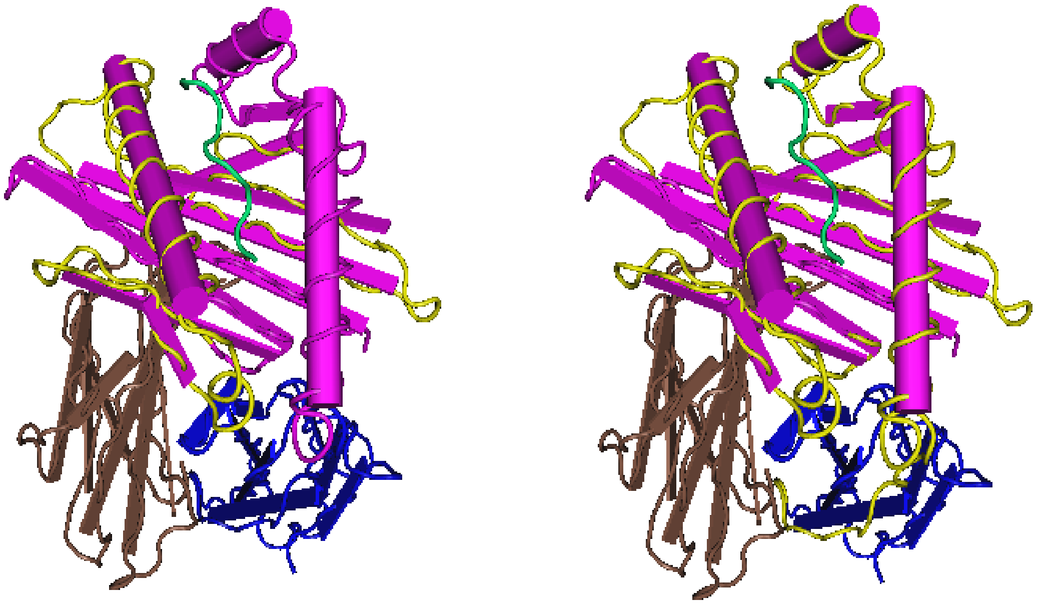

Supplementary figure 1. Molecular 3D structure of the SLA-1 molecule. The binding cleft formed by alpha 1 and -2 domains (pink) are in complex with a peptide (green). The part of the binding cleft covered by the 320 nt. amplicon (left) and 510 nt. amplicon (right) is marked in yellow. The figures are created using the Cn3D version 4.3.1.
