# Supplementary material for: Sequence-Based Genotyping of Expressed Swine Leukocyte Antigen Class I Alleles by Next-Generation Sequencing Reveal Novel Swine Leukocyte Antigen Class I Haplotypes and Alleles in Belgian, Danish, and Kenyan Fattening Pigs and Göttingen Minipigs
Source: Front Immunol. 2017 Jun 16;8:701. doi: 10.3389/fimmu.2017.00701 (PMC5472656; doi:10.3389/fimmu.2017.00701)
Supplement: Supplementary file 6 [file Image_2.PDF]

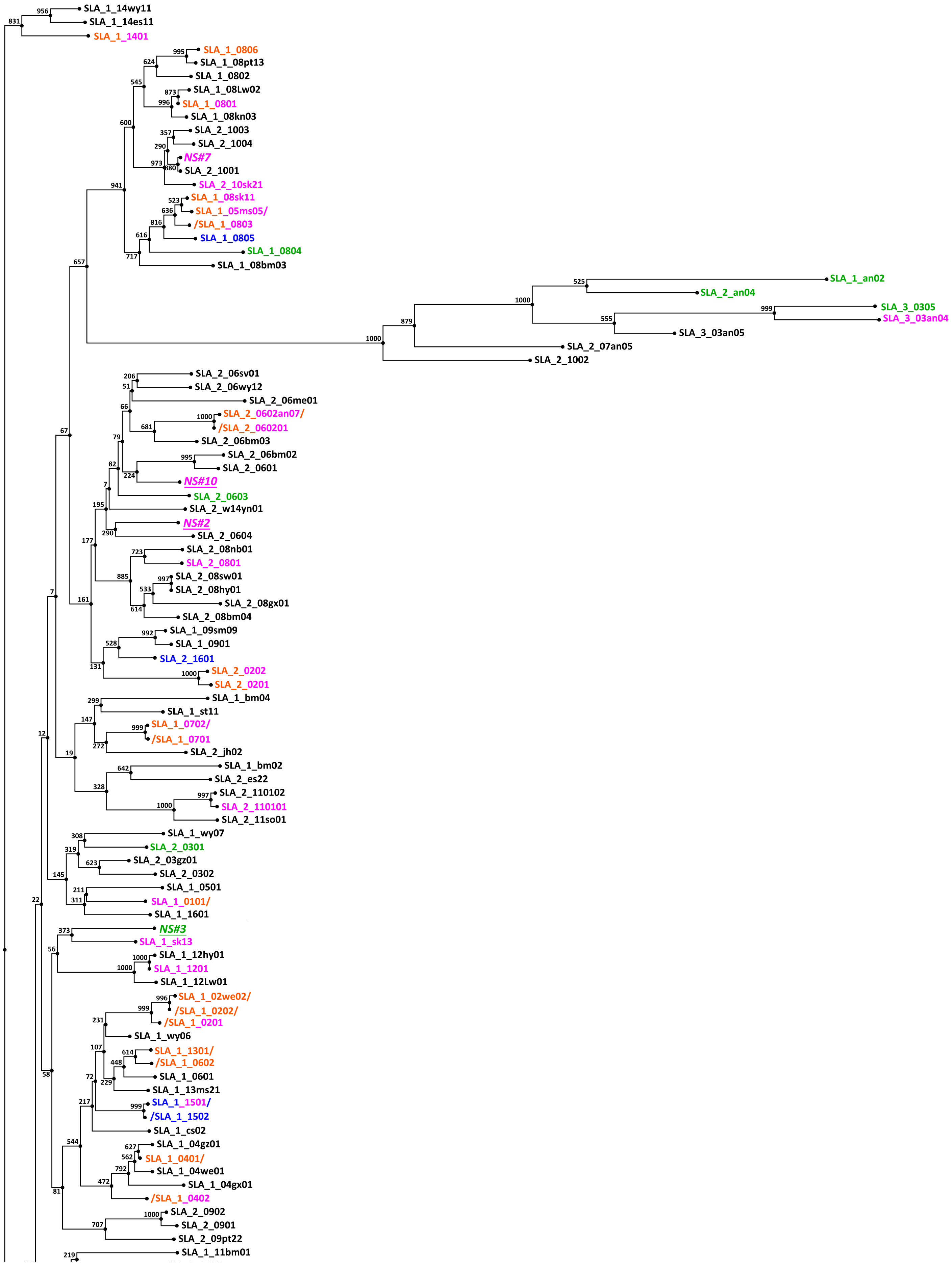
SLA_1_14wy11
SLA_1_14es11
SLA_1_1401
SLA_1_0806
SLA_1_08pt13
SLA_1_0802
SLA_1_08Lw02
SLA_1_0801
SLA_1_08kn03
SLA_2_1003
SLA_2_1004
NS#7
SLA_2_1001
SLA_2_10sk21
SLA_1_08sk11
SLA_1_05ms05/
/SLA_1_0803
SLA_1_0805
SLA_1_0804
SLA_1_08bm03
SLA_1_an02
SLA_2_an04
SLA_3_0305
SLA_3_03an04
SLA_3_03an05
SLA_2_07an05
SLA_2_1002
SLA_2_06sv01
SLA_2_06wy12
SLA_2_06me01
SLA_2_0602an07/
/SLA_2_060201
SLA_2_06bm03
SLA_2_06bm02
SLA_2_0601
NS#10
SLA_2_0603
SLA_2_w14yn01
NS#2
SLA_2_0604
SLA_2_08nb01
SLA_2_0801
SLA_2_08sw01
SLA_2_08hy01
SLA_2_08gx01
SLA_2_08bm04
SLA_1_09sm09
SLA_1_0901
SLA_2_1601
SLA_2_0202
SLA_2_0201
SLA_1_bm04
SLA_1_st11
SLA_1_0702/
/SLA_1_0701
SLA_2_jh02
SLA_1_bm02
SLA_2_es22
SLA_2_110102
SLA_2_110101
SLA_2_11so01
SLA_1_wy07
SLA_2_0301
SLA_2_03gz01
SLA_2_0302
SLA_1_0501
SLA_1_0101/
SLA_1_1601
NS#3
SLA_1_sk13
SLA_1_12hy01
SLA_1_1201
SLA_1_12Lw01
SLA_1_02we02/
/SLA_1_0202/
/SLA_1_0201
SLA_1_wy06
SLA_1_1301/
/SLA_1_0602
SLA_1_0601
SLA_1_13ms21
SLA_1_1501/
/SLA_1_1502
SLA_1_cs02
SLA_1_04gz01
SLA_1_0401/
SLA_1_04we01
SLA_1_04gx01
/SLA_1_0402
SLA_2_0902
SLA_2_0901
SLA_2_09pt22
SLA_1_11bm01

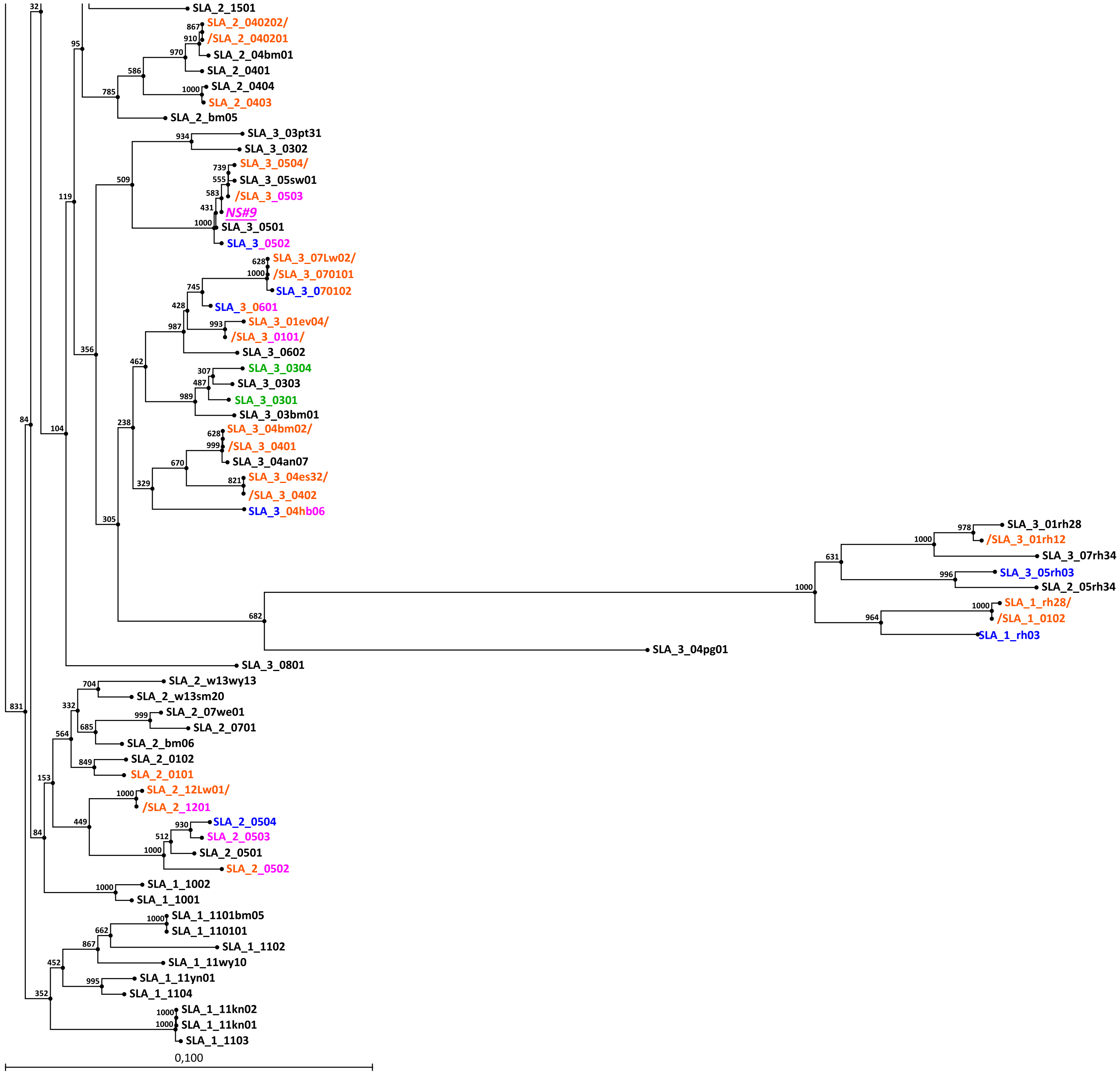

Supplementary figure 2. Phylogenetic analysis of the novel SLA class I sequences. Neighbour joining tree based on 605 nt. covering exon 1 to 4 (nt. 40 – 644) of all known SLA class I alleles and the novel SLA class I sequences discovered in the present study underlined in bold. The tree was constructed using a 1000 bootstraps replicates. Colour codes are used to show in which population the given allele has been found; green: Göttingen minipigs, blue: Kenyan pigs, orange: Danish pigs, and pink: Belgian pigs. The numbers above branches represent bootstrap values. The distances were computed with the Jukes cantor method. The analysis was conducted in CLC genomic workbench 6.5.
